# The Lack of Side Effects of an Ineffective Treatment Facilitates the Development of a Belief in Its Effectiveness

**DOI:** 10.1371/journal.pone.0084084

**Published:** 2014-01-08

**Authors:** Fernando Blanco, Itxaso Barberia, Helena Matute

**Affiliations:** Universidad de Deusto, Departamento de Fundamentos y Métodos de la Psicología, Bilbao, Spain; Federal University of Rio de Janeiro, Brazil

## Abstract

Some alternative medicines enjoy widespread use, and in certain situations are preferred over conventional, validated treatments in spite of the fact that they fail to prove effective when tested scientifically. We propose that the causal illusion, a basic cognitive bias, underlies the belief in the effectiveness of bogus treatments. Therefore, the variables that modulate the former might affect the latter. For example, it is well known that the illusion is boosted when a potential cause occurs with high probability. In this study, we examined the effect of this variable in a fictitious medical scenario. First, we showed that people used a fictitious medicine (i.e., a potential cause of remission) more often when they thought it caused no side effects. Second, the more often they used the medicine, the more likely they were to develop an illusory belief in its effectiveness, despite the fact that it was actually useless. This behavior may be parallel to actual pseudomedicine usage; that because a treatment is thought to be harmless, it is used with high frequency, hence the overestimation of its effectiveness in treating diseases with a high rate of spontaneous relief. This study helps shed light on the motivations spurring the widespread preference of pseudomedicines over scientific medicines. This is a valuable first step toward the development of scientifically validated strategies to counteract the impact of pseudomedicine on society.

## Introduction

In today's knowledge-based society, the wide-spread use and popularity of certain alternative medicines, such as homeopathy, continues to be increasingly troublesome for health authorities worldwide. While rigorous scientific studies have repeatedly shown that homeopathy is completely ineffective (no more effective than placebo [1]), many patients still choose to use it or other pseudomedicines in place of conventional treatments that have been proven effective. This decision results in important consequences, sometimes death [2]. Therefore, one may ask why people prefer to use homeopathy and other alternative medicines over scientifically tested medicines. One possible answer is that the alleged lack of side effects of most alternative medicines (in the case of homeopathy, we assume no side effects because the main ingredient is water) makes the treatment more attractive than conventional scientific medicines, which frequently include unpleasant side effects. While most would agree that people frequently resort to those treatments they believe are more effective, we propose that the reverse also holds: frequent use of a treatment, because of the lack of side effects or other considerations, fuels the belief that it is effective, even when it is not.

It has been previously suggested that a basic cognitive bias, known as the causal illusion, underlies many irrational beliefs, particularly beliefs in the effectiveness of pseudomedicines [3]. The causal illusion is the illusory perception of a contingency between a potential cause and the outcome of interest when they are actually not causally related. In this case, the contingency is between the use of a treatment and the recovery from a disease. A useless treatment is one that does not increase the probability of healing when it is used. That is, the probability of healing remains the same whether or not the treatment is used, P(Healing|Treatment)  =  P(Healing|¬Treatment), and hence the contingency between the two events (treatment and healing) is zero. In recent decades, experimental psychologists have identified a number of conditions that promote the overestimation of zero-contingencies. We argue that the way pseudomedicines are typically used meets the conditions that, according to recent research on causal illusions, facilitate the overestimation of causality, and therefore the belief in the effectiveness of completely useless treatments.

One variable that researchers have identified as a robust facilitator of the causal illusion, at least in controlled experiments, is the probability of the desired outcome. The illusion would appear more prominent when the healings occur with high probability, even if they are not correlated with the use of the treatment [4], [5], [6]. Another variable of interest is the probability of occurrence of the potential cause, P(Cause). Following the example above, P(Cause) is the probability that a patient uses the treatment when she is sick. Thus, in this particular context one can also refer to it as P(Treatment). Basic research suggests that the more often a patient takes a completely useless medicine, the more likely she will develop a belief in its effectiveness. This is particularly true when the desired outcome (the healing) takes place frequently [7], [8].

The effect of P(Cause) on causal judgments has been widely documented in computer-based experiments. These studies are generally theory-focused experiments and could, in principle, make use of any context or scenario, be it real or fictional, in which causal relationships could be assessed by participants. In fact, many of them use medical scenarios as cover-stories in which the potential cause to be evaluated is a fictitious treatment, and the desired outcome is a fictitious patient recovering from a disease [7]. Therefore, the researchers found that P(Treatment) significantly affects the participants' effectiveness judgments of a treatment, at least in these fictitious scenarios. These studies suggest the inversion of what one could take as the obvious relationship between the mentioned variables, P(Treatment) and belief in the effectiveness of the treatment. The obvious relationship is that people’s belief in the effectiveness of a treatment will influence P(Treatment), the probability that they use the treatment. The inverse, less intuitive, relationship is that irrespective of people’s initial expectations, the mere increase in P(Treatment) will increase people’s belief in the effectiveness of the treatment. That is, the experiments suggest that it is the frequent use of the medicine that results in the illusion of its effectiveness.

Moreover, previous experiments show that, when not influenced or instructed by the experimenters, people naturally tend to introduce the target cause (i.e., to use the potential treatment), in more than half of the occasions [7], [9] (see [10] for more general evidence in a neutral scenario). That is, the spontaneous tendency is to use the treatment with high probability, and this normally leads to an illusory perception of effectiveness. Still, it is possible to influence this spontaneous behavior. The manipulation of P(Cause), or P(Treatment), has been achieved in different ways in fictitious experimental scenarios. For instance, (a) by means of explicit instructions about the rate with which the cause should be introduced [8], [10], [11] or (b) by manipulating the availability of the potential cause [12]. These studies showed that increasing P(Cause) facilitates the development of the illusion, while decreasing P(Cause) reduces it. However, the studies directly restricted P(Cause) [12] or at least suggested the adequate P(Cause) that participants should expose themselves to [8], [10]. In this study, our goal was to move one step back and get a glimpse of the actual variability in P(Treatment). We wanted to know which preceding factors determined the probability of using the treatment in real life situations, and whether we could influence P(Treatment) without explicit instructions or limiting the participant's access to the treatment.

In a recent study, Barberia et al. [9] were able to reliably reduce the natural tendency of high school students to introduce a potential cause (i.e., to use a fictitious medicine on a series of fictitious patients) by using an educational intervention explaining the rationale of causal inference, which conforms the basic principle of scientific reasoning and experimental design. In line with this study, we suggest another factor that might indirectly affect the otherwise frequent use of a useless treatment, hence developing a causal illusion of effectiveness. This factor is the presence of side effects produced by the treatment. To our knowledge, this factor has not been manipulated in experimental research. Manipulating this variable may be an effective and very natural way of modulating the cost associated with treatment, and therefore should have an observable impact on the probability of introducing the potential cause (i.e, the treatment). If correct, we would also detect differences in the development of causal illusions, depending on the presence of side-effects.

To sum up, we propose that one of the reasons why people continue to believe in the effectiveness of bogus treatments is their alleged lack of side effects. Because there is no harm in using homeopathy (i.e., the cost associated with using the treatment is very low), it is used frequently. In certain conditions where the base rate of spontaneous remission is high (such as headache, back pain, flu, etc.), the experimental research on contingency learning and causal illusions clearly suggests that introducing the potential cause with high frequency results in overestimation of the effectiveness of the treatment. This would generate a vicious circle in which pseudomedicines are frequently used because they imply low cost (e.g., no harm), and in turn, high rates of pseudomedicine use contributes to an illusory perception of effectiveness, thus reinforcing further consumption.

In our experiment, we adapt the standard contingency learning task that has been used in previous experiments [3] to include a manipulation of the cost associated with using the treatment based on the presence of side effects. Our prediction is that, because a lack of side effects encourages the use of the treatment with high probability, it facilitates the illusory belief that the treatment is working.

## Methods

### Ethics Statement

The ethical review board of the University of Deusto examined and approved the procedure used in this experiment, as a part of a larger research project (Ref: ETK-44/12-13).

### Participants and Apparatus

Seventy-nine anonymous first-year Psychology students from the Universidad Nacional de Educación a Distancia (UNED, Spain) volunteered to take part in the study through the virtual laboratory website [http://www.labpsico.deusto.es] as an optional course activity. No personal information (age, gender) was collected. The computer program assigned each participant to one of two groups, a high-cost group and a no-cost group. Data from five participants were removed because the medicine was not administered to any patient during the session. Thus, the final sample consisted of 74 participants, 39 of whom were in the high-cost group, and 35 in the no-cost group. The experiment was programmed in *JavaScript*, a web-based language that is interpretable by most browsers.

The participants were informed before the experiment that they could quit the study at any moment by closing the browser window. The data collected during the experiment were sent anonymously to the experimenter only upon explicit permission by the participant, indicated by clicking on a "Submit" button. If the participant clicked on the "Cancel" button, the information was erased. No personal information (i.e., name, IP address, e-mail) was collected. In agreement with the ethical guidelines for Internet-based research [13], we did not use cookies or other software to covertly obtain information from the participants.

### Procedure and Design

We adapted a standard contingency learning paradigm that has been extensively used in the literature [14]. Participants were individually presented with a computer task in which they were asked to imagine that they were medical doctors working in an emergency care facility. They were told that crises induced by a dangerous disease called "Lindsay syndrome" should be stopped immediately. Each participant was to heal as many patients as possible. To this end, participants could use a medicine called Batatrim but, since this medicine was still experimental, its effectiveness in treating the disease had not yet been proven. The high-cost group was informed that Batatrim would produce a severe and permanent skin rash as a side effect in every patient who takes it. The no-cost group was not told about any side effect. (An English translation of the full instructions is available as supporting material Instructions S1.)

**Figure 1:**
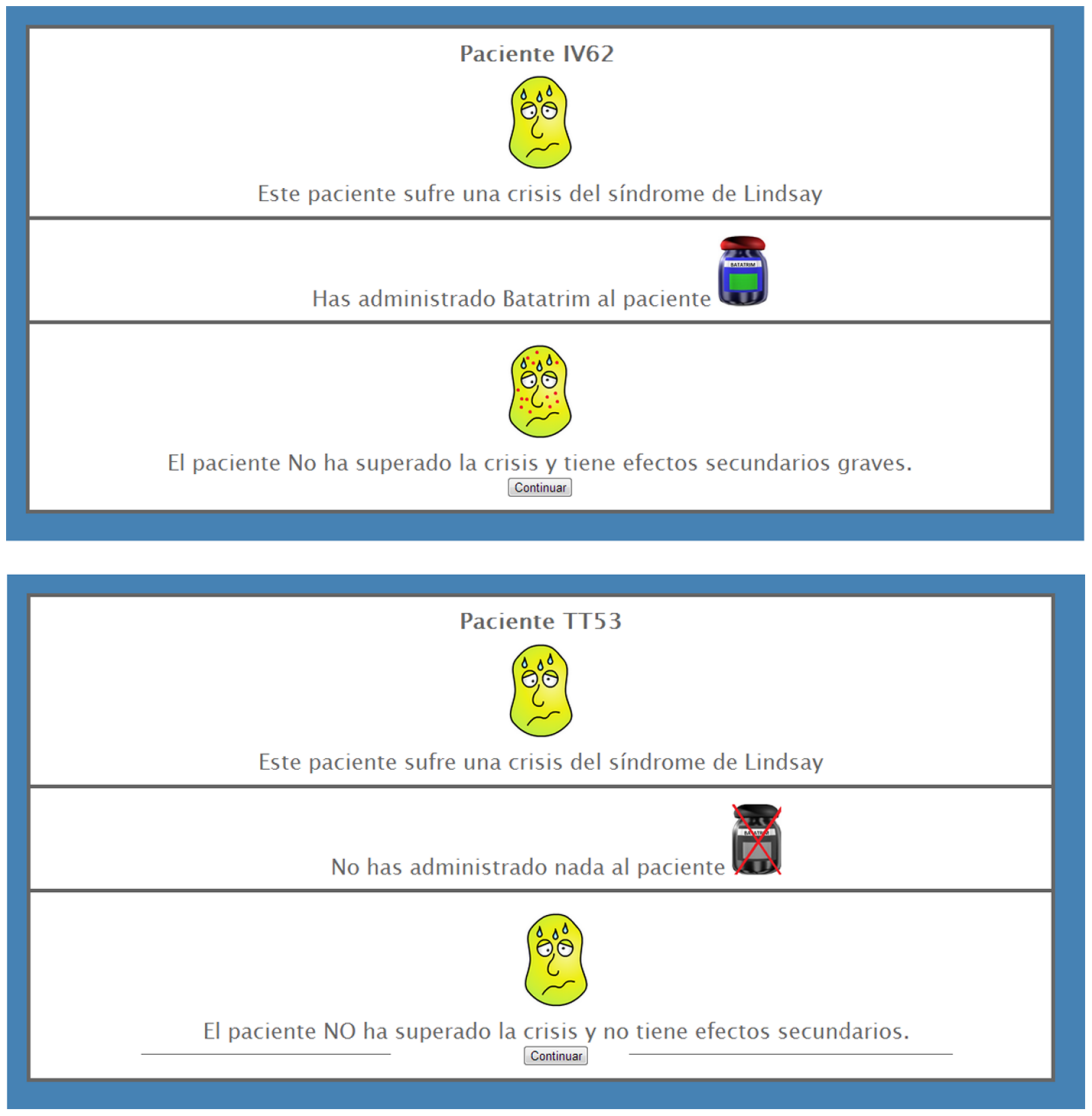
Two samples of the medical records used in the contingency learning task. Medical records were presented sequentially (one new patient per trial). In these two samples, the fictitious patients were programmed to fail to recover. Thus, the outcomes for these two patients show the same symptoms as in the initial state of the trial (greenish skin, sweat) that was always presented in the top panel of each record. The record depicted in the top of the figure corresponds to a patient who was given the medicine by a participant in the high-cost group. The patient developed the skin rash side effect which was added to the symptoms of the syndrome. The record depicted at the bottom of the figure corresponds to a patient in which the participant decided not to use the medicine (the pill bottle is crossed out in red). This patient showed no additional symptoms to the ones provoked by the syndrome alone.

After reading the instructions, 50 medical records of different fictitious patients were presented sequentially. Each record contained a picture showing the patient suffering from the syndrome (i.e., a greenish head covered in beads of sweat) together with a sentence stating that the patient was suffering from a crisis provoked by the syndrome. Immediately below was the question, "*Would you like to give Batatrim to this patient?*" The participant then indicated his decision by clicking on a "Yes" or "No" button. Upon giving the answer, a message indicated whether Batatrim was used by displaying either "*You have given Batatrim to this patient*" or "*You have given nothing to this patient.*" This statement was accompanied by a picture of a medicine bottle, which was crossed out if the participant opted not to use Batatrim. The picture of the sick patient and the indication of Batatrim use were displayed in the top and middle panels, respectively. The bottom panel then displayed the outcome for the patient. Figure 1 shows a sample of two medical records used in the task.

For each participant, in 35 out of 50 trials, the patient recovered from the crisis induced by the syndrome. This outcome was programmed to occur randomly, independent of the participant's decision to use Batatrim. In other words, the outcome took place with high probability (70%) but was programmed to be uncorrelated with use of the medicine.

**Figure 2:**
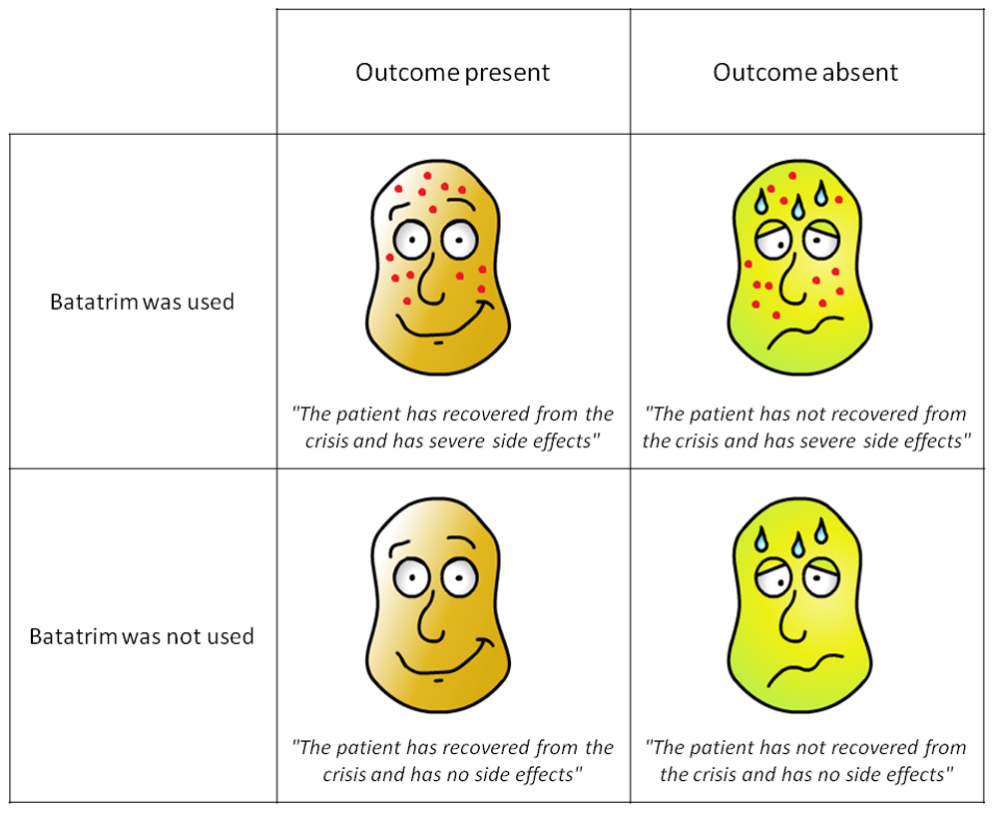
Stimuli used to represent the outcome information in the high-cost group. These consisted of a picture and a message. Each patient either recovered from the crisis (left column) or not (right column). In addition, the cost (side effect) of the action was depicted as a permanent skin rash whenever it was used (top row). The skin rash was never observed otherwise (bottom row). In the no-cost group, regardless the decision to use the medicine, the stimuli were identical to those presented in the bottom row, except for the removal of the reference to side effects in the accompanying messages (i.e., *“The patient has recovered from the crisis”* or *“The patient has not recovered from the crisis”* for the left and right panels, respectively).

In the no-cost group, the outcome was displayed as a picture of a healthy face and the message, "*The patient has recovered from the crisis*", whereas the outcome absence was displayed as a picture of an ill face (greenish, covered in sweat) identical to the one presented in the top panel of the computer screen, and the statement, "*The patient has not recovered from the crisis.*" This procedure and presentation format was identical to those widely used in previous versions of the standard contingency learning task [3]. By contrast, the high-cost group was shown pictures and messages conveying not only the disease outcome, but also the side effects of Batatrim when it was used. Thus, whenever the medicine was given, the picture of the patient showed a skin rash, and the statement also included the words *"...and has severe side effects.*" Likewise, whenever the medicine was not given, the words *"...and has no side effects*" were added to the message. Figure 2 shows a sample of the four stimuli used as outcomes in the high-cost group. Note that the side effects were described and visually depicted as different from the symptoms produced by the Lindsay syndrome crisis; the former were presented as a skin rash of red spots covering the head, and the latter as a combination of greenish skin color and beads of sweat. The symptoms of the syndrome, identical in all the patients, were always visible in the top panel of the screen, which showed the initial state of the patient. The side effects, when they appeared, were superimposed on the symptoms of the illness in the lower panel of the screen. This allowed visual comparison between the initial and final status of the patient, with the aim of preventing participants from confusing the patient's symptoms and the medicine’s side effects.

At the end of the experiment, the participants were asked to rate the perceived effectiveness of Batatrim by asking, "To what extent do you think that Batatrim has been effective to stop the crises of Lindsay syndrome in the patients you have just seen?" They indicated their rating by clicking on a numerical scale from zero to 100 where zero was "It has been completely ineffective to stop the crises," 50 was "It has been moderately effective to stop the crises," and 100 was "It has been perfectly effective to stop the crises." Because the recoveries from the syndrome occurred equally often in the presence and in the absence of the medicine, the higher the effectiveness ratings, the stronger the overestimation of the zero contingency between the medicine and the recoveries.

## Results

**Figure 3:**
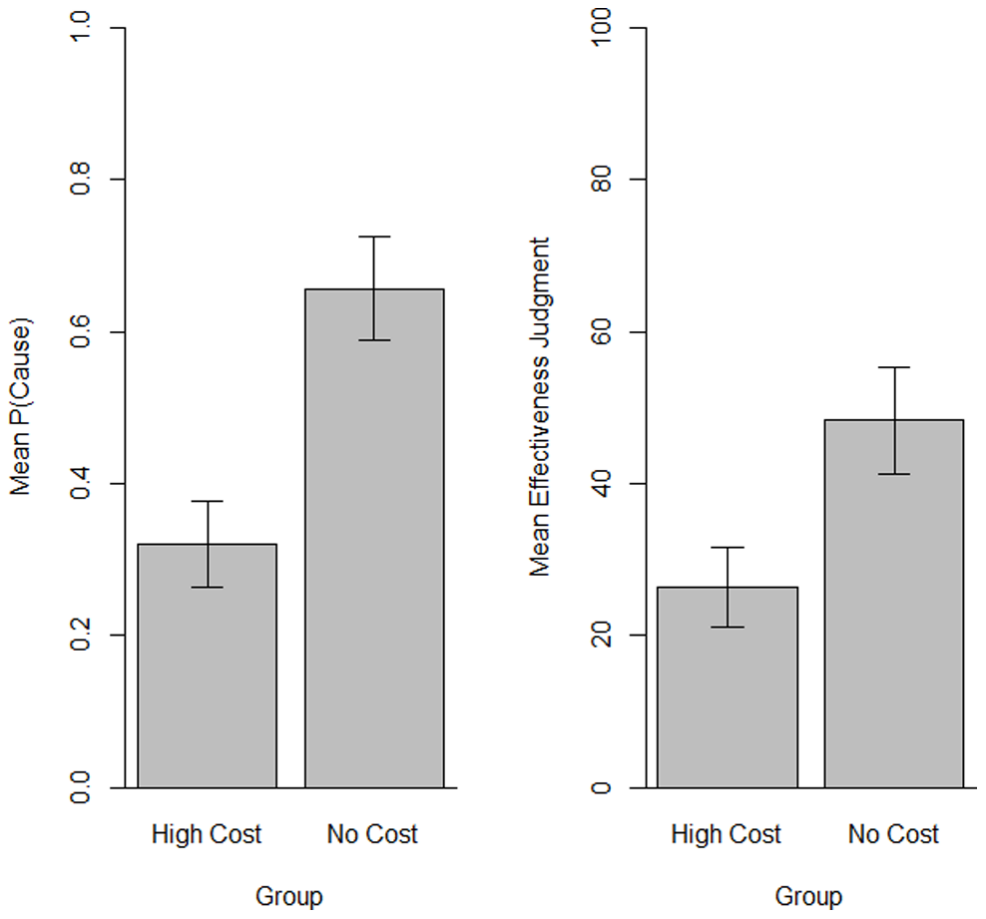
Results of the experiment. The left panel shows the mean probability of introducing the potential cause, P(Cause), in each of the two groups. The right panel shows the mean effectiveness judgments given by participants in the two groups. Error bars depict 95% confidence intervals for the means.

The left panel in Figure 3 depicts the mean probability of using Batatrim, i.e., the P(Cause), for each group. A one-way ANOVA indicated that, as expected, P(Cause) was significantly higher in the no-cost group than in the high-cost group, *F*(1, 72)  = 23.87, *p*<0.001, η_p_^2^ = 0.25. The no-cost group gave the treatment to more than 50% of the patients, *t*(34)  = 2.08, *p*<0.005 in agreement with previous research showing a spontaneous tendency to use the medicine with high probability. The high-cost group gave the treatment to significantly fewer than 50% of the patients, *t*(38)  = 3.86, *p*<0.001. The mean effectiveness judgments given by the participants at the end of the session were also higher in the no-cost group, *F*(1, 72)  = 10.64, *p*<0.005, η_p_^2^ = 0.13 (Figure 3).

We were also interested in testing whether the effect of the cost manipulation on the effectiveness judgments could be attributed to the mediator role of P(Cause). This mediational hypothesis has received empirical support [9], [11] concerning the effectiveness of a fictitious medicine. We wished to test whether it held true for our case, where the cost of administering the medicine, rather than an instructional manipulation or an educational workshop, was what prompted the participants to reduce P(Cause).

**Figure 4:**
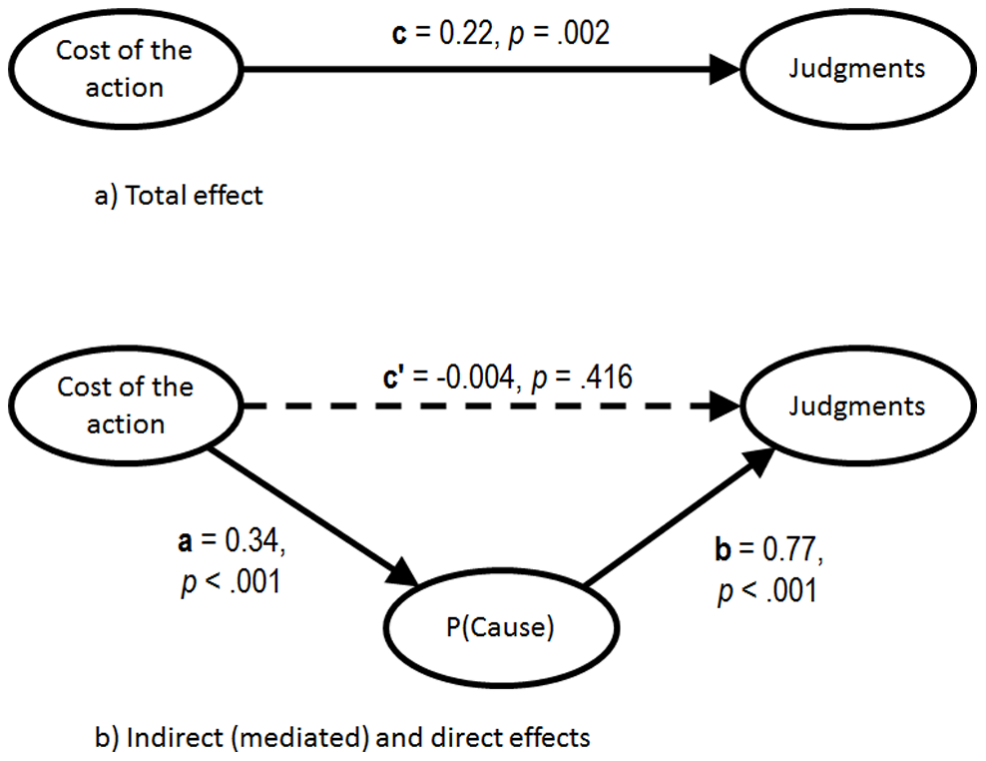
Mediational structure underlying the experimental manipulation. The total effect of the cost of the action on the effectiveness judgments, depicted as path c (top panel), is partitioned into two components, one indirect effect through P(Cause) (paths a and b, bottom panel), and one direct effect (path c', bottom panel), which is the result of discounting the indirect effect. The unstandardized coefficients and p-values for each pathway are provided. These results suggest that the effect of the cost associated with the treatment, manipulated between groups, was completely mediated by P(Cause).

According to the mediational structure hypothesis, the total effect of the cost manipulation on the judgments was composed of two effects, one direct effect and one indirect effect through P(Cause) (Figure 4). These effects were assessed using the procedure described by Hayes [15]. The resulting (unstandardized) coefficients are shown in Figure 4. The total effect of the cost manipulation on effectiveness judgments was significant, *c* = 0.22, *t*(73)  = 3.26, *p*<0.005, 95% CI = 0.08 to 0.35, and partitioned into two pathways (Figure 4) that were examined in turn. The direct effect of the cost manipulation, *c*' = −0.04, *t*(72)  = −0.82, *p* = 0.42, 95% CI = −0.13 to 0.06, was not significant once it was controlled for P(Cause). The indirect effect was composed of two pathways, both of which were significant, *a* = 0.34, *t*(73)  = 4.89, *p*<0.001, 95% CI = 0.20 to 0.47, and *b* = 0.77, *t*(72)  = 10.76, *p*<0.001, 95% CI = 0.63 to 0.91. The former shows a strong relationship between cost manipulation and P(Cause) and the latter, a strong positive relationship between P(Cause) and effectiveness judgments even after controlling for the effect of the cost manipulation. These results suggest, as expected, that P(Cause) completely mediated the effect of the cost manipulation on the effectiveness judgments.

Recent evidence [15] recommends that inferences about the indirect effect are based not on the significance of the individual paths (*a* and *b*), but rather, on the explicit quantification of the indirect path itself. This was achieved by using the PROCESS macro [15]. We employed the bias-corrected bootstrap resampling method (5000 samples) made available by this tool to compute 95% confidence intervals for the indirect effect of the cost manipulation through P(Cause) (i.e., pathway a*b). The analyses confirmed the mediator role of P(Cause), a*b = 0.26, 95% CI = 0.15 to 0.36, in that the entire confidence interval was above zero. In addition, the proportion of the total effect due to the indirect effect was 1.18, 95% CI = 0.83 to 2.20, further strengthening support for the total mediation hypothesis.

## Discussion

In this study, we have shown that knowing a medicine produces side effects prevented the overestimation of its effectiveness that is typically observed when the percentage of spontaneous remissions is high [7], [10]. We demonstrated that the mechanism by which this effect works rests on the lower frequency of the treatment usage exhibited by those participants who were aware of the medicine's side effects.

Previous work in the more general domain of causal illusions made use of a procedure similar to the one we employed in the no-cost group in that, they did not mention any side effect of the medicine. As a result, they found a spontaneous tendency to use the treatment with relatively high frequency [7], [9], and, consequently, a strong overestimation of the effectiveness of the medicine. The cost manipulation that we implemented via the explicit inclusion of side effects demonstrated how these illusions can be readily reduced, and reinforced the idea that the causal illusion is strongly determined by P(Cause) as previous research suggests [7], [8], [9], [10].

We therefore assumed that the effect of manipulating the side effects in this study is completely attributable to subsequent changes in P(Cause). Further research is needed to study the mechanism linking high P(Cause) and the illusory perception of causality. One possible explanation for this P(Cause) effect rests on the increase in the number of coincidences between the potential cause (i.e., the use of the medicine) and the outcome (i.e., the recovery from the disease). Because both the potential cause and the outcome happen very frequently, they are likely to coincide accidentally [10]. These fortuitous pairings lead to the development of an illusory causal link between them, and thus to an irrational effectiveness overestimation, similar to a superstition. This was inspired by early precedents, namely, the adventitious reinforcement designs reported by Skinner in 1948 [16] (although Skinner's interpretations of his data as "superstitious behavior" were later criticized [17], recent advancements converge to support the role of accidental coincidences in the development, by reinforcement, of systematic behavior patterns even in laboratory studies using animal subjects [18]). The coincidence-based account for the P(Cause effect) is readily accommodated by current leading theories proposed to explain causal and associative learning. For instance, associative theories such as the Rescorla-Wagner model [19] predict the illusion of causality as long as many cause-outcome coincidences occur early in the experiment. Similarly, several alternative causal learning models, based on statistical rules, predict the illusion because they weigh more heavily on the trials in which these coincidences occur [20], [21]. In any case, the relationship between P(Cause) and the illusion of causality is a matter of theoretical discussion in the associative and causal learning field, and goes beyond the focus of our work (see [7] for further discussion on this point).

Importantly, the introduction of the high cost of using the treatment renders our experimental task more natural and ecological than the standard human contingency learning paradigms commonly used in causal illusion research, in which no cost of the action is imposed. A psychological experiment is most often an artificial situation where little information is given and few behavioral options are available to the participant. In contrast, a typical real life situation involves a wide variety of factors affecting human decisions and behaviors. These factors may be economic cost, effort, magnitude of benefit, competing alternatives, etc. While our side-effect manipulation arguably made the task more ecological than paradigms commonly used in the literature, one must remain cautious when extending our results to real pseudomedicine use, since many of the above-mentioned factors are outside the scope of our simplified experimental setting. Once this limitation is acknowledged, we believe it is plausible to assume that our results are applicable to pseudomedicine use in several ways.

Most pseudomedicines are used in conditions that promote the illusion of efficacy, and, as seen in our experiment, these alternative treatments are more often applied to mild diseases with a high rate of spontaneous remission (headache, back pain, etc.) This parallels experiments conducted where there is a high probability of the desired outcome, resulting in strong overestimations of zero contingencies [4], [5], [6]. In addition, many pseudomedicines are advertised as harmless, as opposed to most conventional treatments, which typically produce undesired side effects. As shown in our experiment, the lack of side effects increased the probability of the cause (i.e., the frequency with which the medicine is prescribed), which in turn is known to be another factor that results in overestimations of zero contingencies [7], [8], [10], that frequently used medicines will be more likely considered effective even if they are completely useless. Moreover, when both a high probability of the outcome and a high probability of the cause are combined, the chances that the two events coincide accidentally increase, and therefore the causal illusion is strongly facilitated [5], as predicted by leading theoretical accounts of causal learning. It can be safely predicted that, once a treatment is considered at least somewhat effective, it will be used even more frequently, resulting in higher chances of further accidental coincidences. This feeds a vicious circle in which accidental occurrences of a desired outcome reinforce actions that are thought to produce them.

If the knowledge acquired from the experimental research on causal illusions and causal learning can be used to better understand why certain pseudomedicines are popular in the public, it can also be valuable in overcoming problems derived from the belief in the effectiveness of these treatments. Our first recommendation for the developers of educational interventions to prevent or combat these beliefs is that they should stress the benefit of using scientifically validated treatments over the benefit of avoiding side effects. If the patient's focus is on the latter point, she will generally start using the pseudomedicine because of the low cost associated with this action (e.g., there is no harm in using it). We have documented that this is likely to result in an illusion of effectiveness. Our second recommendation is to draw the patient's attention to the occasions in which the use of the medicine and health improvement do not co-occur; either the treatment is not followed by the healing, or the healing occurs without taking the pseudomedicine. Because people spontaneously grant more credit to response-outcome coincidences [22] and different theories agree in the crucial role that these coincidences posses in the genesis of causal illusions, reminding people to pay attention to the events in which treatment and healing occur separately could prevent or reduce the illusion. This approach was adopted in a recent intervention designed for high school students described by Barberia et al. [9]. Their intervention was successful in reducing the tendency to develop causal illusions, as measured by means of a contingency learning task very similar to the one used here. To summarize, we believe that the knowledge obtained in basic experimental psychology can fruitfully contribute to prevent and eradicate certain widespread harmful behaviors and beliefs.

## Supporting Information

Instructions S1 **Full instructions of the experiment (translated from the original in Spanish).** The underlined sentences were omitted from the no-cost group.
(PDF)
