# Supplementary material for: The Lack of Side Effects of an Ineffective Treatment Facilitates the Development of a Belief in Its Effectiveness
Source: PLoS One. 2014 Jan 8;9(1):e84084. doi: 10.1371/journal.pone.0084084 (PMC3885525; doi:10.1371/journal.pone.0084084)
Supplement: Instructions S1 — Full instructions of the experiment (translated from the original in Spanish). The underlined sentences were omitted from the no-cost group. (PDF) [file pone.0084084.s001.pdf]

Appendix

Full instructions (translated from the original in Spanish). The underlined sentences were omitted from the no-cost group.

Imagine that you are a doctor who works at the Hospital Emergency Department. You are a specialist in a rare and dangerous disease called "Lindsay Syndrome", which must be treated quickly in the emergency room.

Crises induced by this illness may be stopped immediately by using a medicine called "Batatrim", but this medicine is still in its testing stage, and therefore its reliability has not been yet proven. In addition, this medicine can produce severe side effects. Specifically, a permanent and very annoying skin rash that can last for the rest of their lives.

Now, you will be presented with a series of medical records of patients suffering from Lindsay Syndrome. In each record, you will see a patient and you will have the opportunity of either using Batatrim or not. Then, you will know whether or not the patient actually recovered from the crisis and also whether or not he/she developed side effects due to Batatrim use. Try to make that as many patients as possible recover from the Lindsay Syndrome crises. Once you have observed a number of patients, you will be asked a few questions.
